# Workplace health and safety training, employees’ risk perceptions, behavioral safety compliance, and perceived job insecurity during COVID-19: Data of Vietnam

**DOI:** 10.1016/j.dib.2020.106346

**Published:** 2020-09-24

**Authors:** Hsinkuang Chi, Thinh-Van Vu, Tan Vo-Thanh, Nguyen Phong Nguyen, Duy Van Nguyen

**Affiliations:** a Department of Business Administration, Nanhua University, Taiwan; b Thuongmai University, Hanoi, Vietnam; c Excelia Business School – CEREGE (EA 1722) – France; d School of Accounting, University of Economics Ho Chi Minh City, Ho Chi Minh City, Vietnam; e Quantitative Analysis Center, Hanoi, Vietnam

**Keywords:** COVID-19, Workplace health and safety training, Risk perceptions, Safety compliance, Job insecurity

## Abstract

This paper presents the dataset of a survey on workplace health and safety training, employees’ risk perceptions, behavioral safety compliance, and perceived job insecurity in Vietnam during COVID-19 pandemic. The data were collected through an online questionnaire completed by Vietnamese full-time employees between April and June 2020. Using E-mail, LinkedIn, and Facebook, the online questionnaire was sent to respondents who filled it out voluntarily. A two-wave survey was conducted in order to lessen the common method bias. Totally, we received complete matched data for 732 full-time employees. All data were processed through SPSS 22.0, AMOS 23.0 and Smart PLS 3.0. Besides descriptive statistics, the results of the explanatory factor analysis and the confirmation factor analysis were included in this paper, which may serve as a good reference for future studies.

## Specifications Table

**Table:** 

Subject	Business, Management and Accounting
Specific subject area	Human resource management, Workplace health and safety management, Risk perceptions, Employee perception and behavior.
Type of data	Table
How data were acquired	Survey Questionnaire (included in Supplementary Materials)
Data format	Raw Analyzed
Parameters for data collection	Respondents are Vietnamese employees who participated in the survey voluntarily
Description of data collection	The data were collected through an online questionnaire completed by Vietnamese full-time employees between April and June 2020. Using E-mail, LinkedIn, and Facebook, the online questionnaire was sent to respondents who filled it out voluntarily. A two-wave survey was conducted in order to lessen the common method bias. Totally, we received complete matched data for 732 full-time employees.
Data source location	Region: Asia Country: Vietnam
Data accessibility	Mendeley depository Direct URL: http://dx.doi.org/10.17632/8b3hrcykcr.1

## Value of the Data

• This dataset advances the knowledge regarding the impact of workplace health and safety training on employees’ perceived risk of COVID-19, their behavioral safety compliance at the workplace, and perceived job insecurity.
• The present data is particularly useful for organizational behavior and human resource management researchers and organization managers to understand employees’ perceptions and behavior during the pandemic.
• The data can be reused for an empirical study that intends to examine workplace health and safety practices and employees’ attitudes and behaviors during the COVID-19 pandemic in Vietnam, compared with other countries.
• The dataset is a reference source for studies on workplace health and safety management as well as human resource management during a health crisis.

## 1 Data Description

COVID-19 originated in Wuhan, China, in December 2019, and became a global pandemic [1,2], the worst global crisis since the Second world war [3]. During this pandemic, workplace health and safety training should be provided to all levels of employees to improve their awareness, knowledge, and attitudes to health and safety in the workplace. Previous studies demonstrated that employees’ awareness of the risks associated with the pandemic could influence their attitudes and behaviors [4,5]. According to the protection motivation theory [6], behavior adjustment may be achieved by playing to people's fears. Therefore, workplace health and safety training pandemic could have an impact on employees’ perceived risk of COVID-19, which, in turn, influences their behavioral safety compliance and their perceived job insecurity.

The questionnaire included two main information sections: socio-demographic- and work-related information and measurement scales. Concretely, the first section consisted of information related to respondent's characteristics, including age (4 categories), gender (2 categories), position (4 categories), type of work contract (3 categories), size of working organization (7 categories) and type of working organization (3 categories), organization type (5 categories), industry (6 categories), and working mode change due to COVID-19 (4 categories: constant, switch to working at home completely, about a half of work being done at home, and a small part of work being done at home). To fully complete the form, respondents spent about 12 min. Seven hundred thirty-two valid responses were collected through a two-wave survey. Respondents’ profiles are shown in Table 1.

**Table 1:** Respondents’ profiles (*N* = 732).

	*N*	%		*N*	%
***Age***			***Gender***		
<30	343	46.9	Male	337	46.0
30–40	258	35.2	Female	395	54.0
41–50	99	13.5	**Position**		
>50	32	4.4	Employee	503	68.7
***Type of work contract***			First-line chief	104	14.2
1-year contract or shorter	76	10.4	Middle manager	89	12.2
Contract from over 1 year to 3 years	74	10.1	Top manager	36	4.9
Permanent contract	258	35.2	***Organization size (employees)***		
***Organization type***			< 50	161	22.0
State administrations	183	25.0	51–100	106	14.5
State company	38	5.2	101–200	88	12.0
Domestic private company	285	38.9	201–500	87	11.9
Foreign-invested company	148	20.2	501–1000	147	20.1
Others	78	10.7	1001–2000	58	7.9
***Industry***			> 2000	85	11.6
Manufacturing or processing	185	18.7	***Working mode change due to COVID-19***		
Tourism and hospitality services	178	18.0	Constant (any change)	273	37.3
Warehousing and logistics	58	5.9	Switch to working at home completely	197	26.9
Education	240	24.3	About a half of work being done at home	170	23.2
Trade, wholesale, and retail	141	14.4	A small part of work being done at home	92	12.6
Others	185	18.7			

The second section consisted of items related to workplace health and safety training (5 items, Cronbach's alpha = 0.911), employees’ risk perceptions of COVID-19 (6 items, Cronbach's alpha =0.72), employees’ behavioral safety compliance (5 items, Cronbach's alpha = 0.925), and their perceived job insecurity during COVID-19 pandemic (5 items, Cronbach's alpha = 0.88) (see Table 2). We provided the results of exploratory factor analysis (EFA) with SPSS software, demonstrating that the 21 items were saliently loaded onto four dimensions, namely, workplace health and safety training, risk perceptions of COVID-19, behavioral safety compliance, job insecurity. The analysis was grounded on relevant ratios such as Kaiser-Meyer-Olkin statistic (KMO) equal to or higher than 0.50, Barlett test with p-value smaller than 0.05, and average variance extracted over 50%, factor loadings of each item of more than 0.50 (see Table 2).

**Table 2:** Descriptive and exploratory factor analysis results.

Variable		Mean	SD	Factor loadings in the EFA			
				*HST*	*PC*	*SC*	*JI*
*Workplace health and safety training (HST) (Cronbach's alpha = 0.91)*							
HST1	My company gives comprehensive training to employees in workplace health and safety issues	3.53	1.16	.865			
HST2	All employees must participate in training programs on COVID-19 prevention	3.31	1.26	.865			
HST3	Training programs on COVID-19 prevention given to me are adequate to enable me to assess hazards in the workplace	3.80	1.12	.849			
HST4	Management promotes internal communication on COVID-19 prevention via newsletter, e-mail, Facebook, etc.	4.14	1.03	.732			
HST5	Safety issues are given high priority in training programs	4.06	1.05	.762			

*Risk perceptions of COVID-19 (PC) (Cronbach's alpha =0.72)*							
PC1	COVID-19 has a high fatality rate	4.34	0.94		.676		
PC2	Currently, the treatment methods for COVID-19 are not effective	2.92	1.17		.787		
PC3	We will need to wait for a long time before having a vaccine for COVID-19	3.13	1.18		.768		
PC4	I am worried about myself, my family members or my colleagues who may be affected by COVID-19	4.24	1.00		.763		
PC5	I believe it is possible that there will be an outbreak of COVID-19 in the area where I live and work	3.31	1.15		.648		
PC6	In general, I know that COVID-19 is highly dangerous	4.50	0.87		.786		

*Behavioral safety compliance (SC) (Cronbach's alpha = 0.93)*							
SC1	I use all the necessary safety equipment (masks, hand washing products, etc.) to prevent COVID-19	4.51	0.80			.801	
SC2	I respect safety rules and procedures regarding the prevention of COVID-19 while carrying out my job	4.47	0.80			.851	
SC3	I ensure the highest levels of safety when I carry out my job.	4.45	0.79			.862	
SC4	I carry out my work in a safe manner.	4.30	0.88			.815	
SC5	I do not deviate from correct and safe work procedures	4.33	0.86			.842	

*Perceived job insecurity (JI) (Cronbach's alpha = 0.88)*							
JI1	I am worried about having to quit my job before I would like to due to COVID-19.	3.38	1.37				.626
JI2	There is a risk that I will have to leave my current job in the near future.	2.73	1.40				.785
JI3	My career development opportunities in the organization are favorable. (R)	2.39	1.26				.829
JI4	I feel that the organization can provide me with a stimulating job content in the near future. (R)	2.36	1.24				.793
JI5	I believe that the organization will still need my competence in the future even if the COVID-19 pandemic breaks out. (R).	2.11	1.21				.818
JI6	My salary, bonus, and other benefits will still be promising in the near future even if the COVID-19 breaks out. (R)	3.00	1.37				.735
JI7	I am afraid that my salary, bonus, and other benefits development will be delayed due to COVID-19.	2.99	1.30				.749

Note: (R) indicates that the item was reverse coded. SD = Standard deviation.

Afterward, we validated the measure of the constructs through confirmatory factor analysis (CFA) with AMOS 23.0 and Smart PLS 3.0. In this analysis, the items PC2–3 of risk perceptions of COVID-19 and item JI1 of perceived job insecurity are eliminated because of their standardized factor loadings less than 0.50. The CFA justifies that the model fit indices meet the acceptable criteria [7] as shown that *χ*^2^ = 512.913 (*df* =160, *χ*^2^/*df* = 3.026, *p* < 0.001), RMSEA = 0.055 < 0.08, RMR = 0.075 < 0.08, GFI = 0.931 > 0.9; CFI = 0.963 > 0.9, and TLI = 0.956 > 0.9. In addition, in order to check the reliability and the convergent validity of the measurement model, we computed the average variance extracted (AVE) and composite reliability (CR) values [8]. Table 3 presents that these constructs had AVE values greater than the 0.50 cut-off (from 0.565 to 0.77), and CR over 0.70 (from 0.831 to 0.944). Moreover, most of the outer loadings were above 0.50, except that of the item PC5, with a value of 0.438. We decided to retain PC5 as its outer loading was not smaller than 0.40 to be deleted as suggested by Avkiran and Ringle [9]. Thus, the measurement model was considered reliable. We then checked the measurement model for discriminant validity using the procedure suggested by Fornell and Larcker [10]. Table 4 shows that the square root values of AVE (bold diagonal) of the constructs (ranging between 0.751 and 0.878) were all higher than the absolute values of their correlations (between 0.131 and 0.533). This result shows an adequate level of discriminant validity. We also calculated the Heterotrait-Monotrait (HTMT) ratios to further confirm the discriminant validity and found the result was as robust as the HTMT ratios, ranging between 0.118 and 0.566, and were significantly less than 0.85 [11].

**Table 3:** Confirmation factor analysis results.

Constructs and items		Weight/ loading
*Workplace health and safety training (HST) (Cronbach's alpha = 0.911; AVE = 0.734; CR = 0.932)*		
HST1	My company gives comprehensive training to employees in workplace health and safety issues	0.848
HST2	All employees must participate in training programs on COVID-19 prevention	0.808
HST3	Training programs on COVID-19 prevention given to me are adequate to enable me to assess hazards in the workplace	0.872
HST4	Management promotes internal communication on COVID-19 prevention via newsletter, e-mail, Facebook, etc.	0.869
HST5	Safety issues are given high priority in training programs	0.884

*Risk perceptions of COVID-19 (PC) (Cronbach's alpha = 0.751; AVE = 0.831; CR = 0.565)*		
PC1	COVID-19 has a high fatality rate	0.805
PC4	I am worried about myself, my family members or my colleagues who may be affected by COVID-19	0.793
PC5	I believe it is possible that there will be an outbreak of COVID-19 in the area where I live and work	0.438
PC6	In general, I know that COVID-19 is highly dangerous	0.889

*Behavioral safety compliance (SC) (Cronbach's alpha = 0.925; AVE = 0.944; CR = 0.770)*		
SC1	I use all the necessary safety equipment (masks, hand washing products, etc.) to prevent COVID-19	0.868
SC2	I respect safety rules and procedures regarding the prevention of COVID-19 while carrying out my job	0.899
SC3	I ensure the highest levels of safety when I carry out my job.	0.897
SC4	I carry out my work in a safe manner.	0.853
SC5	I do not deviate from correct and safe work procedures	0.870

*Perceived job insecurity (JI) (Cronbach's alpha = 0.885; AVE = 0.904; CR = 0.617)*		
JI2	There is a risk that I will have to leave my current job in the near future.	0.715
JI3	My career development opportunities in the organization are favorable. (R)	0.893
JI4	I feel that the organization can provide me with a stimulating job content in the near future. (R)	0.904
JI5	I believe that the organization will still need my competence in the future even if the COVID-19 pandemic breaks out. (R).	0.902
JI6	My salary, bonus, and other benefits will still be promising in the near future even if the COVID-19 breaks out. (R)	0.609
JI7	I am afraid that my salary, bonus, and other benefits development will be delayed due to COVID-19.	0.624

Note: (R) indicates that the item was reverse coded.

**Table 4:** Discriminant validity analysis.

	Mean	SD	HST	PC	SC	JI
1. Workplace health and safety training (HST)	3.768	0.964	**0.857**			
2. Perceived job insecurity (JI)	2.518	1.054	−0.131⁎⁎ *0.118*	**0.785**		
3. Risk perceptions of COVID-19 (PC)	4.095	0.744	0.345⁎⁎ *0.368*	−0.039⁎⁎ *(0.172)*	**0.751**	
4. Behavioral safety compliance (SC)	4.410	0.725	0.533⁎⁎ *0.566*	−0.16⁎⁎ *0.133*	0.438⁎⁎ *0.459*	**0.878**

*Note*: 1st value = Correlation between variables (off diagonal); 2nd value (italic) = HTMT ratio; SD = Standard deviation; Square root of average variance extracted (bold diagonal).

⁎⁎ : Correlation is significant at the 1% level (2-tailed *t*-test).

## 2 Experimental Design, Materials, and Methods

Four primary constructs in this survey were measured using scales extracted from previous studies. These scales were adapted to the context of COVID-19. Specifically, the scales of workplace health and safety training (5 items) and employees’ behavioral safety compliance (5 items) were adapted from Vinodkumar and Bhasi [12]; the scale of employees’ risk perceptions (6 items) was adapted from Lau et al. [13]; the scale of employees’ perceived job insecurity (7 items) was adapted from Hellgren et al. [14]. The questionnaire was translated into Vietnamese and then back into English to avoid changes in meaning. We followed the recommendation of Hardesty and Bearden [15] by inviting eight experts specialized in human resource management (two full professors, four assistant professors, and two Ph.D. students) from three universities. The experts read the constructs’ items adapted and provided us with suggestions to guarantee face validity. As a result, all the items were agreed by 75% or more of the experts and some wordings were adjusted. Moreover, to ensure the readability, the Vietnamese version was tested on five Vietnamese full-time employees and refined based on their feedback. The survey instrument consisted of 31 questions, including 23 statements of specific impact of COVID-19 which require participants to rate on a 5-point Likert scale, particularly 1 = Totally disagree; 2 = Somewhat disagree; 3 = Neither agree nor disagree; 4 = Somewhat agree; 5 = Totally agree.

The data were collected through an online questionnaire completed by Vietnamese full-time employees between April and June 2020. Using E-mail, LinkedIn, and Facebook, the online questionnaire was sent to respondents who filled it out voluntarily. We preferred this data collection method to reduce the risks of infection for participants and researchers. A two-wave survey was conducted in order to lessen the common method bias [16]. We set a cover letter at the beginning of the questionnaire indicating the survey objective and the procedure of this survey, assuring respondents about the confidentiality of their data. At wave 1, respondents reported their socio-demographic information, E-mails, and workplace health and safety training, employees’ risk perceptions of COVID-19. At this stage, we collected 917 respondents. Data on dependent variables (employees’ behavioral safety compliance and perceived job insecurity) were collected at wave 2 after 10 days. A short time frame between phase 1 and phase 2 was chosen to reduce the drop rate and memory bias [17]. The two-wave data were matched through an identification code assigned to each respondent. The use of the codes allowed us to exclude participants’ email addresses, ensuring the confidential nature of the survey. Totally, we received complete matched data for 732 full-time employees. All data were processed through SPSS 22.0, AMOS 23.0 and Smart PLS 3.0.

## Ethics Statement

Before data collection, the instrument was reviewed and approved by the Ethics Committee of the University of Economics Ho Chi Minh City (No: 1661/QD-DHKT-QLKH). The authors received informed consent from participants. Participation was voluntary, and they could withdraw from the survey at any point. As an ethical research team, we value the privacy rights of human subjects. Therefore, the data we submitted does not identify participants based on their responses. The survey did not collect any identifiable information from the participants.

## Declaration of Competing Interest

The research team did not receive financial support from any institutions. The authors declare that they have no known competing financial interests or personal relationships that could have appeared to influence the work reported in this paper.

## Appendix Supplementary materials

Image, application 1
